# AC Pulsed Field Ablation Is Feasible and Safe in Atrial and Ventricular Settings: A Proof-of-Concept Chronic Animal Study

**DOI:** 10.3389/fbioe.2020.552357

**Published:** 2020-12-03

**Authors:** Guido Caluori, Eva Odehnalova, Tomasz Jadczyk, Martin Pesl, Iveta Pavlova, Lucia Valikova, Steffen Holzinger, Veronika Novotna, Vladimir Rotrekl, Ales Hampl, Michal Crha, Dalibor Cervinka, Zdenek Starek

**Affiliations:** ^1^International Clinical Research Center, St. Anne’s University Hospital Brno, Brno, Czechia; ^2^IHU LIRYC, Electrophysiology and Heart Modeling Institute, Fondation Bordeaux Université, Pessac, France; ^3^Univ. Bordeaux, INSERM, UMR 1045, Cardiothoracic Research Center of Bordeaux, Pessac, France; ^4^Department of Cardiology and Structural Heart Diseases, Medical University of Silesia, Katowice, Poland; ^5^Department of Biology, Faculty of Medicine, Masaryk University, Brno, Czechia; ^6^First Department of Internal Medicine-Cardioangiology, St. Anne’s University Hospital, Masaryk University, Brno, Czechia; ^7^Institute of Scientific Instruments of the Czech Academy of Sciences, Brno, Czechia; ^8^Faculty of Veterinary Medicine, University of Veterinary and Pharmaceutical Sciences, Brno, Czechia; ^9^R&D EP Systems & Sensors, BIOTRONIK SE & Co., KG, Berlin, Germany; ^10^Department of Power Electrical and Electronic Engineering, Faculty of Electrical Engineering and Communication, Brno University of Technology, Brno, Czechia; ^11^Department of Histology and Embryology, Faculty of Medicine, Masaryk University, Brno, Czechia

**Keywords:** pulsed field ablation, irreversible electroporation (IRE), radiofrequency ablation, atrial fibrillation, ventricular arrhythmia (VA), preclinical cardiology

## Abstract

**Introduction:**

Pulsed field ablation (PFA) exploits the delivery of short high-voltage shocks to induce cells death via irreversible electroporation. The therapy offers a potential paradigm shift for catheter ablation of cardiac arrhythmia. We designed an AC-burst generator and therapeutic strategy, based on the existing knowledge between efficacy and safety among different pulses. We performed a proof-of-concept chronic animal trial to test the feasibility and safety of our method and technology.

**Methods:**

We employed 6 female swine – weight 53.75 ± 4.77 kg – in this study. With fluoroscopic and electroanatomical mapping assistance, we performed ECG-gated AC-PFA in the following settings: in the left atrium with a decapolar loop catheter with electrodes connected in bipolar fashion; across the interventricular septum applying energy between the distal electrodes of two tip catheters. After procedure and 4-week follow-up, the animals were euthanized, and the hearts were inspected for tissue changes and characterized. We perform finite element method simulation of our AC-PFA scenarios to corroborate our method and better interpret our findings.

**Results:**

We applied square, 50% duty cycle, AC bursts of 100 μs duration, 100 kHz internal frequency, 900 V for 60 pulses in the atrium and 1500 V for 120 pulses in the septum. The inter-burst interval was determined by the native heart rhythm – 69 ± 9 bpm. Acute changes in the atrial and ventricular electrograms were immediately visible at the sites of AC-PFA – signals were elongated and reduced in amplitude (*p* < 0.0001) and tissue impedance dropped (*p* = 0.011). No adverse event (e.g., esophageal temperature rises or gas bubble streams) was observed – while twitching was avoided by addition of electrosurgical return electrodes. The implemented numerical simulations confirmed the non-thermal nature of our AC-PFA and provided specific information on the estimated treated area and need of pulse trains. The postmortem chest inspection showed no peripheral damage, but epicardial and endocardial discolorations at sites of ablation. T1-weighted scans revealed specific tissue changes in atria and ventricles, confirmed to be fibrotic scars via trichrome staining. We found isolated, transmural and continuous scars. A surviving cardiomyocyte core was visible in basal ventricular lesions.

**Conclusion:**

We proved that our method and technology of AC-PFA is feasible and safe for atrial and ventricular myocardial ablation, supporting their systematic investigation into effectiveness evaluation for the treatment of cardiac arrhythmia. Further optimization, with energy titration or longer follow-up, is required for a robust atrial and ventricular AC-PFA.

## Introduction

Pulsed field ablation (PFA) is a recent method for interventional treatment of cardiac arrhythmia. It exploits the localized application of short – nano/microseconds - high-voltage electric fields to induce irreversible electroporation, a prolonged state or pore-induced permeability that triggers non-necrotic deaths (Davalos et al., 2005), in the proximal tissue (Wittkampf et al., 2018; Wojtaszczyk et al., 2018; Sugrue et al., 2019) with high selectivity (Miklavčič, 2018) and without strict need of contact (Ramirez et al., 2020). These characteristics make the method an excellent candidate to avoid thermal collateral damages observed with radiofrequency and cryo-balloon ablation, among which phrenic/vagal nerve injury/palsy, atrio-esophageal fistula, pulmonary vein stenosis and thrombus formation (Sugrue et al., 2018; Wojtaszczyk et al., 2018).

Different tissues present different sensitivities to irreversible electroporation, with the striated muscle (including myocardium) proven the most susceptible one (Wittkampf et al., 2018), for reasons still not fully understood. This peculiar sensitivity prompted researchers and physicians to perfect a selective ablation of heart muscle using pulsed irreversible electroporation. Several short-term animal studies have proven how PFA does not affect the coronary vasculature, the local innervation, and the adjacent structures (e.g., esophagus and lungs) (Sugrue et al., 2019). A first in-human trial for paroxysmal atrial fibrillation ablation via pulmonary vein isolation has delivered promising results, with a 12-month Kaplan-Meier estimate of freedom from arrhythmia of 87.4 ± 5.6%, outperforming in the medium term the mentioned radiofrequency (RF) and cryo-balloon studies (Reddy et al., 2019).

Notwithstanding these promising reports, several open issues call for more investigation and optimization. There is no consensus on which pulse shape and dynamics possess the best tradeoff between efficacy and safety. Pulsed electric field energy can be delivered according to different parameters: Shape of the voltage pulse, predominantly DC/AC square; voltage level and current compliance; number of pulses; interpulse distance (Wojtaszczyk et al., 2018).

DC monophasic pulses are historically the most employed for electroporation of cell suspensions, but they can lead to potentially painful nerve and muscle capture (Mercadal et al., 2017), arcing, metallic release in solution (Meir and Rubinsky, 2014), and bubble stream formation, also due to electrolysis without arcing (van Es et al., 2019a). These issues require a careful control of DC pulses delivery and avoid direct galvanic coupling between electrodes and tissue. AC biphasic pulses or bursts can help to prevent these issues (Wojtaszczyk et al., 2018), although they impose a lower equivalent electric field on the tissue, and they are more subjected to electrode surface heating due to skin-effect. Asymmetrical AC has been introduced, proved feasible and effective in animal studies (Stewart et al., 2019) and clinical trials (Reddy et al., 2018). Both symmetrical and asymmetrical AC pulses have been proved to ablate ventricular myocardium in an epicardial porcine model with customized applicators (van Es et al., 2019b). From this evidence, symmetrical AC bursts of sufficient duration do not suffer from “cancelation effect”, where the effect of the first phase of the pulse is canceled by the second phase (Polajžer et al., 2020). Therefore, AC bursts are a suitable PFA waveform yet to be tested in endocardial settings with commercial catheters.

The voltage level is determinant to impose a sufficiently high electric field across the tissue – hundreds of Vcm^–1^. The available catheter technology and cardiac anatomy require pulse generators to provide at least units of kV. Current compliance, typically in tens of A, is determined by the quality of tissue/electrode contact and the number of electrode pairs to excite in parallel: this determines the design of the pulse generator components with respect to the application. Multiple pulse delivery is preferable to keep a longer state of enhanced permeability to favor irreversible electroporation-related processes, although it might lead to heating and overtreatment (Garcia et al., 2014). Interpulse distance, especially in cardiac applications, is conditioned by synchronization with the ventricular effective refractory period – an approach a.k.a. ECG-gating – not to elicit potentially lethal arrhythmias (Arena et al., 2011).

Given this set of knowledge, we have designed a pulse delivery strategy for PFA with a purely AC square burst, timed by ECG-gating. In our developed generator, a transformer-based design provides excellent insulation between the power line and the electrodes applied in the patients’ heart. We have envisioned two uses of our pulse generator: Parallel energy delivery through a circular catheter for pulmonary vein isolation; two-catheter energy delivery across the ventricular septum to test ventricular arrhythmia ablation. We have tested our implemented technology and design of PFA on a chronic swine model.

## Materials and Methods

### Animal Preparation

The animal experiments were approved by the ethic commission of the University of Veterinary and Pharmaceutical Sciences in Brno. We used 6 female swine – weight 53.75 ± 4.77 kg, 6 months old. One animal was used in a pilot procedure primarily to identify the: maximum voltage applicable in each setting; the optimal circular catheter size, in terms of navigation and multiple application in the left atrium. All the operators and the supporting staff were protected from X-ray radiation during procedures, and potentially dangerous chemicals were handled according to safety norms.

Anesthesia and preparation were performed accordingly to previously published studies (Soucek et al., 2020). Briefly, the animals were generally anesthetized and kept under mechanical ventilation with 1.5% isoflurane. We shaved the chest and groin, where we applied limb and precordial ECG electrodes. Two venous and two arterial access were obtained on the groins using 8F introducers. Blood pressure was monitored via one arterial access on the groin. Electroanatomical mapping patches (CARTO, Biosense Webster, Irvine, CA, United States) were placed on the back and the chest according manufacturer instructions. One electrosurgical return electrode was placed, to avoid muscle twitching, on the lower back and connected to the grounding pole of the AC-PFA generator.

Amiodarone (5 mg/kg) was administered to prevent ventricular fibrillation. Heparin (10000 Units) was administered in bolus after introducers and catheters insertion and was continued with half the initial dose every hour.

### Mapping and Ablation

A 6F diagnostic quadripolar catheter (Inquiry^TM^, Abbott, Chicago, IL, United States) was inserted in the right ventricle for monitoring and subsequently act as reference electrode in ventricular ablation. We obtained a left atrial access via transseptal puncture assisted with an ultrasound catheter (AcuNav^TM^, Biosense Webster). On the left side we used a steerable sheath (Agilis^TM^, Abbott) to insert and guide a 7F decapolar circular catheter (AFocus II^TM^, Abbott) or an 8F force-sensing, 4 mm tip ablation catheter (Thermocool^®^ Smarttouch^®^, Biosense Webster). We obtained anatomical and voltage mapping with the ablation catheter, with irrigation 2 ml/min and contact force > 5 g.

We performed PFA in the left atrium aiming at the following locations: right and left pulmonary vein ostia; lateral, anterior and anteroseptal walls. In the ventricular compartment, we ablated the following locations: apical and basal interventricular septum with AC bursts; anterolateral wall with radiofrequency energy – 30 W, 20 s, 30 ml/min – provided by a commercial generator (Smartablate^TM^, Biosense Webster). A custom ECG-gating circuit controlled the AC-burst delivery by detecting the over-threshold voltage of the QRS complex on lead III of the surface ECG. The pulses were delivered in sinus rhythm, with heart rate measured of 69 ± 9 bpm. Each AC burst had a square shape, duty cycle 50%, duration of 100 μs and internal high frequency 100 kHz (i.e., ten oscillations per burst).

**FIGURE 1:**
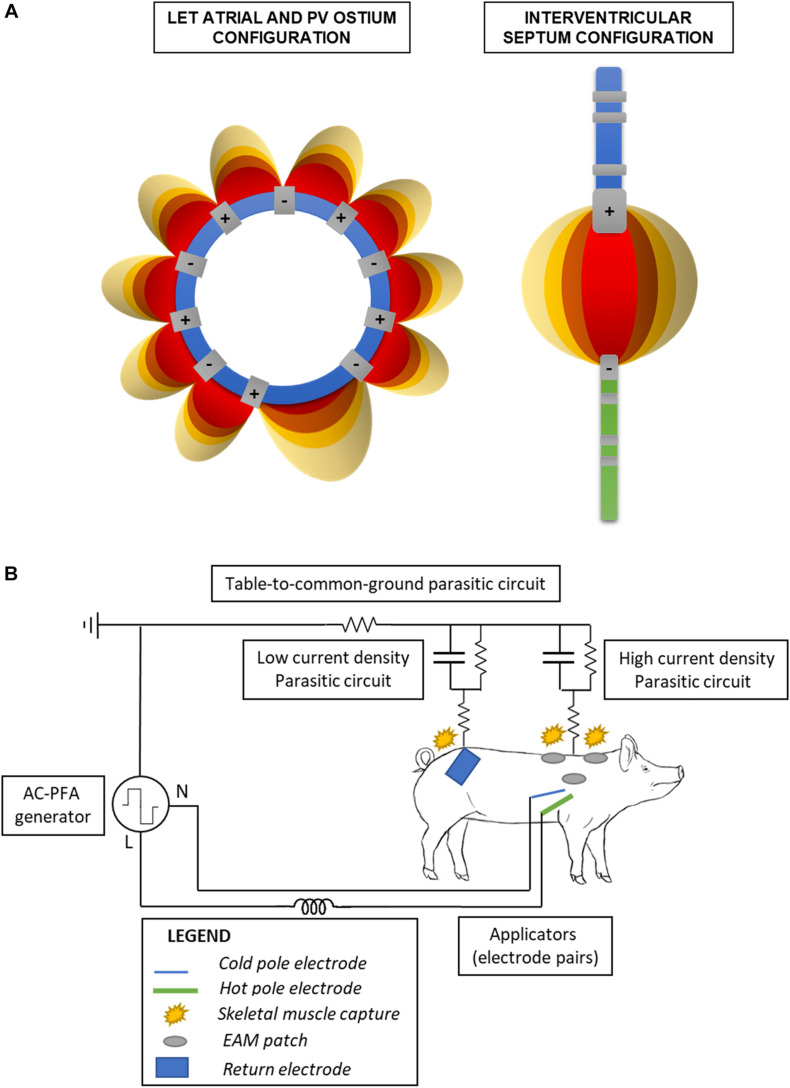
Schematic of the electronics configuration. **(A)** Bipolar catheter electrodes pairings in the atrial and ventricular configuration. The electric fields internal to the loop (left) are not shown for clarity of the figure. **(B)** Simplified schematic of the electric circuits formed in the operating table, when employing the ventricular configuration. Parasitic circuits are present through the table to the common ground, and the return electrode favors a low impedance/current density pathway on the lower back avoiding capture of the upper limbs into muscle twitching.

The AC-PFA generator (registered at the Czech Industry Propriety Office, utility model no. 33133; Czech patent 308415 approved 28/07/2020) was connected to the common ground. Using a custom-made switchbox (Supplementary Figure 1A), we connected the odd number electrodes of the circular catheter to the generator line pole while the even numbers to the neutral pole; this allowed us to have consecutive and shifting anode-cathode pairs across the loop (Figure 1A). We used the same box to connect the ablation catheter distal electrode to the line pole and the diagnostic catheter one to the neutral pole. A detailed schematic of the relevant electrical circuits present in the operating room is presented in Figure 1B. Electrode contact before ablation was overall verified via fluoroscopy and catheter positions on the electroanatomical maps.

For safety reasons, we used a 3-probe esophageal temperature probe set behind the animal’s heart to measure eventual temperature spikes during atrial applications. We also employed intracardiac echo to observe an eventual bubble streaming flowing out of the aortic valve.

After the procedure, the animals were awakened, underwent antibiotic and analgesic therapy, and recovered for 4 weeks. In two animals, we repeated the electroanatomical maps to monitor eventual changes in the voltage amplitude of the treated tissue. All the animals were eventually euthanized by overdose of T61 anesthetic and they underwent pathological inspection and characterization of tissue changes.

### Tissue Inspection, Magnetic Resonance Imaging and Histopathology

After euthanasia, we opened the chest cavity to inspect the heart status and the eventual peripheral damages. The organ was excised and inspected, before perfusion fixation in 10% formaldehyde. The organs were fixed for at least 1 week prior to magnetic resonance imaging.

The samples were scanned as a whole and then divided in atria and ventricles. To provide a control for the tissue changes in the atria, a non-ablated left atrium was excised and scanned from an age-matched animal involved in a parallel trial on ventricular ablation. Before scanning, the samples were rinsed with physiological salt solution (0.9% w/v NaCl), to remove all formaldehyde residues, and placed in a saline-filled jar. Air bubbles inside the samples were carefully removed to avoid imaging artifacts.

We scanned the samples with a 9.4T magnetic resonance system (Bruker BioSpec 94/30USR), equipped with volume coil 1H 198/154 mm, as previously described (Jež et al., 2019) with few modifications. The methods are detailed in the Supplements. Briefly, T2-weighted images were obtained in axial, sagittal and coronal direction using a TurboRARE sequence. T1-weighted images were obtained from the atria parts in axial direction only using a T1-FLASH sequence. The scans covered the whole sample volumes with a slice thickness of 1mm, no gap between slices and up to 512 × 512 pixel resolution. The scientist performing the scans was aware of the protocol name and pig number, but not aware of the specific treatment received. The scans were converted into DICOM series, segmented for volumetric calculations and rendered using ITK Snap (Yushkevich et al., 2006). The images were analyzed by a researcher with 4-year experience in RF ablation lesion evaluation. The image analysis was not blinded because of the presence of a single treatment group and control. The percentage amount of non-myocardial atrial tissue, estimating the amount of native and ablation-derived non-muscular tissue, was calculated as:

(1) $$V_{\%}^{i}=\frac{V_{tot}^{i}-V_{not\_myo}^{i}}{V_{tot}^{i}}$$

Where $V_{tot}^{i}$ is the volume of the specimen *i* = 1–5, and $V_{not\_myo}^{i}$ is the volume of tissue presenting a T1 different from the myocardial one, in the *i*^*th*^ specimen. The final ablated volume was estimated with the formula:

(2) $$V_{\%}^{i^{*}}=V_{\%}^{i}-V_{\%}^{0}$$

Where $V_{\%}^{0}$ is the percentage of non-myocardium present in an age-matched non-ablated atrium. As further individual-specific estimator, we have calculated the ratio between the ablated volume in an individual atrium, and the cylindrical volume obtained by extruding the loop catheter along the thickest section of the atrial wall. After scanning, selected atria and ventricles were sliced, paraffin-embedded and stained with hematoxylin/eosin or trichrome staining for histopathological examination. We digitized the tissue slides with a TissueFAX system (TissueGnostics GmbH, Wien, Austria), 600 × 600 μm field of view and 200x magnification. The expert technician confirming the presence of fibrosis was blinded about the different treatments producing the lesions and was only aware on the animal number and the original location (left atrium or interventricular septum). The linear dimensions of the detected fibrosis -width and depth - were measured in ImageJ (Rueden et al., 2017) after image calibration. Again, the image analysis was not blinded because of univocal origin of the fibrotic lesions, with RF used as control for qualitative tissue changes and not size.

### Finite Element Method AC-PFA Numerical Simulations

To validate our ablation design, we performed finite element simulations in COMSOL Multiphysics 5.5. The blood flow is simulated by a static simulation of laminar flow solving the Navier-Stokes equations. The result of this static simulation is then inserted in a dynamics simulation using the electric currents interface of the AC/DC module coupled to the bioheat transfer interface of the heat transfer module. The single AC-burst had the same aforementioned features used *in vivo*.

We implemented two models for the left atrium scenarios: ablation across a pulmonary vein ostium, with diameter ranging 12–14 mm; ablation of the atrial wall, free of trabecule. To simulate the ventricular scenarios, we considered the two catheters across a 15 mm myocardium barrier in 3 configurations: perfect alignment; 10 mm alignment offset; 45° relative tilting; a combination of the previous two. The complete set of simulated geometries are shown in Supplementary Figure 3A–F. The simulated geometries were meshed by a free tetrahedral method.

The biophysical parameters for myocardium and blood are taken from the literature and are listed in Supplementary Table 1. All other material properties of the catheter (Pt electrodes and Nylon as insulator material) are taken from the COMSOL database.

### Statistical Evaluation

The continuous variables are represented as mean ± standard deviation. Confirmation of normal distribution, statistical tests and graphs are obtained in GraphPad Prism 8 or Matlab. For comparisons between two groups we employed the paired Student’s t-test with Welch’s correction when necessary. For comparisons among multiple groups we employed the Kruskal-Wallis test with Dunn’s *post hoc* correction. A p-value inferior to 0.05 was considered significant.

## Results

### Ablation Procedure and Safety

The pilot animal died of complications due to transseptal puncture and did not complete the follow-up. The remaining five animals underwent the programmed AC-PFA protocol. The ECG-gating circuit timed the AC-bursts delivery in the effective refractory period of the QT interval, with delays ranging 50–75 ms (Supplementary Figure 1B). The voltage level was maximized, limited by the 12 A current compliance of our AC-PFA generator, to V_*atria*_ = 900 V and V_*ventricle*_ = 1500 V. An example of the pulse shape is visible in Supplementary Figure 1C.

**FIGURE 2:**
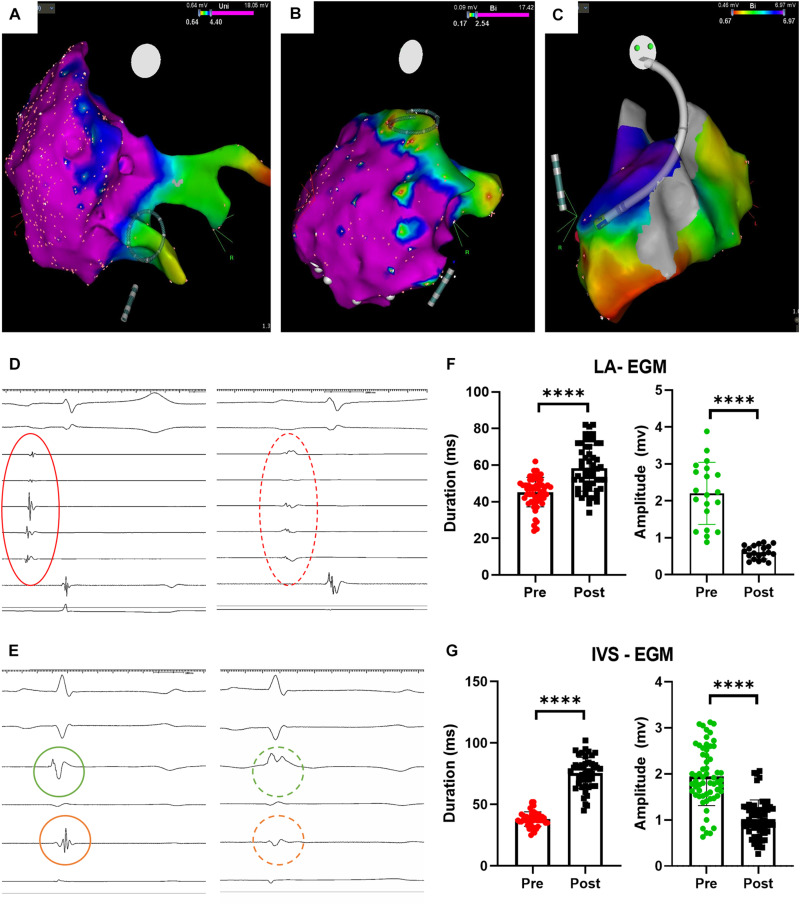
Procedural imaging and electrophysiological results. **(A)** Circular catheter positioning before pulmonary vein ostium ablation; **(B)** Circular catheter positioning before atrial wall ablation; **(C)** catheter positioning across the interventricular septum before ablation; **(D)** atrial electrograms before (red continuous circle) and after (red dashed circle) ablation; **(E)** ventricular electrograms before (green, on left ventricle, and orange, on right ventricle, continuous circle) and after (dashed circles) ablation; **(F)** atrial electrograms changes upon ablation (****p < 0.0001); **(G)** ventricular electrograms changes upon ablation (****p < 0.0001). LA = left atrium; IVS = interventricular septum, EGM = electrogram.

We successfully maneuvered toward the pulmonary vein ostia, atrial walls and interventricular septum (Figure 2A–C). Not all planned ablations were possible in all the animals due to different atrial size, difficulty in outlining the pulmonary veins, or timing. We applied 60-burst trains in the atrium and 120-burst ones across the interventricular septum, accounting for differences in the tissue volume to be treated. The esophageal probe did not show temperature increase during applications; the intracardiac echography did not detect a gas bubble stream formation; we observed occasional twitching of the upped limbs during atrial applications, in absence or bad contact of the return electrode (Supplementary Video 1).

After each AC-burst train application, we monitored significant changes in the intracardiac electrograms (Figure 1D for atria and Figure 1E for ventricles). We quantified differences in electrograms duration – elongated after ablation - and amplitude – decreased after ablation - of the atrial and ventricular electrograms. Occasionally, the ventricular electrogram had an only positive shape, with no changes on the surface ECG. These results are detailed in Table 1. We measured the impedance changes upon ablation, finding a significant decrease (-31.75 ± 13.92 Ω @100 Hz, *p* = 0.011).

**TABLE 1:** Electrophysiological parameters changes upon ablation.

Application	EGM amplitude pre-ablation (mV)	EGM amplitude post-ablation (mV)	EGM duration pre-ablation (ms)	EGM duration post-ablation (ms)	*p*-value
**Left atrium**	2.20 ± 0.84	0.61 ± 0.18	45.29 ± 8.1	58.38 ± 12.61	<0.0001
**Interventricular septum**	1.95 ± 0.63	1.02 ± 0.41	38.20 ± 5.894	75.62 ± 12.70	<0.0001

At the end of procedure, no charring or clots were observed on the catheter surface (Supplementary Figure 2A). We noticed in the first and second animal burned areas in correspondence of the mapping electrodes on the back (Supplementary Figure 2B,C). The burnt regions healed in a week, causing no visible discomfort to the animals. This collateral damage was avoided in the next animals by placing one return electrodes on their back, thus increasing the surface for dissipation of parasitic currents.

### AC-PFA Numerical Simulations

We performed atrial and ventricular simulations imposing a voltage level of 900V and 1500V, respectively, as these were the maximum voltage levels imposable *in vivo*. The single AC-burst had the same duration and high frequency features of the one used *in vivo*. We used a static flow simulation of the atrial wall applications with a circular catheter as a worst-case scenario to simulate temperature rise in tissue. Upon the delivery of an AC burst, the simulation showed a temperature spike which followed an exponential decay. We fitted the simulated curves and found the following parameters: amplitude peak A_*blood*_ = 19°C and A_*tissue*_ = 7.6°C; decay constant τ_*blood*_ = 2.91 ms and one τ_*tissue*_ = 9.22 ms. Due to the considered interpulse interval of 800 ms – as determined by a resting 75 bpm sinus rhythm – we concluded that the temperature rise, and other physical parameters, can be studied with a single AC burst.

We then tested, on the same model, the effect of the convective cooling effect of parallel blood flow: the blood temperature peak dropped from 56.5°C (no flow) to 47.5°C (0.08 ms^–1^ and above). A two-fold velocity increase above 0.08 ms^–1^ determined a 1% temperature decrease. We concluded that, in conditions of normal flow, where the pulmonary venous velocity is approximately double in late diastole (0.16 ± 0.09 ms^–1^) (Meijburg et al., 1992), the AC-burst we use does not drive a temperature increase sufficient to elicit a thermal ablation (Haines, 1993; Dewhirst et al., 2003).

**FIGURE 3:**
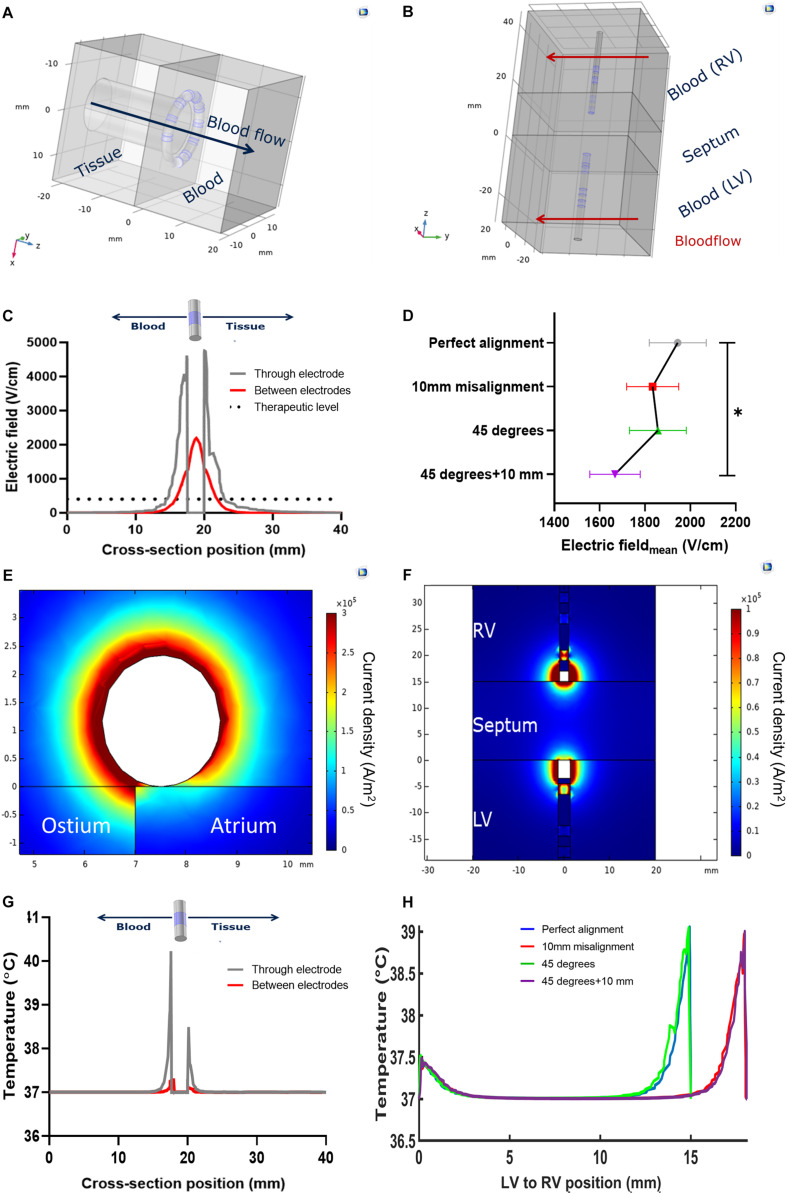
Models and notable results of numerical simulations. **(A)** Geometry of the pulmonary vein ostium ablation; **(B)** Geometry of the interventricular septum ablation, with perfect alignment; **(C)** Electric field variation across the circular catheter used in the left atrium; **(D)** Average electric field across the interventricular septum, with respect to the relative linear misalignment or angular tilting (*p < 0.05); **(E)** current density profile in pulmonary vein ostium ablation; **(F)** current density profile in the interventricular septum ablation in perfect alignment; **(G)** Temperature profile across the circular catheter used in the left atrium; **(H)** temperature profile across the interventricular septum upon ablation, with respect to the relative linear misalignment or angular tilting.

The electrothermal simulations of the atrial (Figure 3A) and ventricular (Figure 3B) ablations show the electric field sharply decreasing from the electrode surface. An electric field with enough strength to elicit irreversible electroporation in muscle (above 400 Vcm^−1^) (Èoroviæ et al., 2010; Kaminska et al., 2012) is present in a 3 mm radius ca. from the electrode surface. This radius decreases to approximately 1.85 mm in the space between the electrodes (Figure 3C). In the ventricular model, in conditions of perfect alignment, the effective electric field radius was 5 mm from the electrode surfaces. This radius was slightly affected by the inspected alignment errors: we estimated an 8.8% coefficient of variation. The average electric field strength decreased significantly when the electrode pairs were misaligned and tilted (*p* = 0.044, Figure 3D). The untreated region was wider when the two electrodes were misaligned, irrespective of tilting – from 4 mm without misalignment to 8 mm ca (Supplementary Table 2).

The current density in all the models was higher in the blood pool, showing an asymmetric profile in the catheters’ cross sections (Figures 3C,D). Correspondingly with the different electrode size, in the ventricular models the current density was higher at the diagnostic distal electrode. The temperature at the tissue-electrode interface was slightly over 40°C. In the ventricular models, this was higher at the diagnostic distal electrode interface. Some notable results from the simulations are shown in Table 2.

**TABLE 2:** Notable results of the electrothermal simulations.

Model	Voltage applied (V)	Electric field max (kV/cm)	Electric field mean (V/cm)	Current density mean (kA/m^2^)	Current density max (kA/m^2^)	Temperature max at the interface (°C)
**Atrial wall**	900	5.19	0.95	47.32	361.52	40.11
**Pulmonary vein ostium**	900	2.19	0.48	40.37	317.55	40.19
**Interventricular septum**	1500	4.84	1.97	56.01	127.57	39.13

### Tissue Changes

**FIGURE 4:**
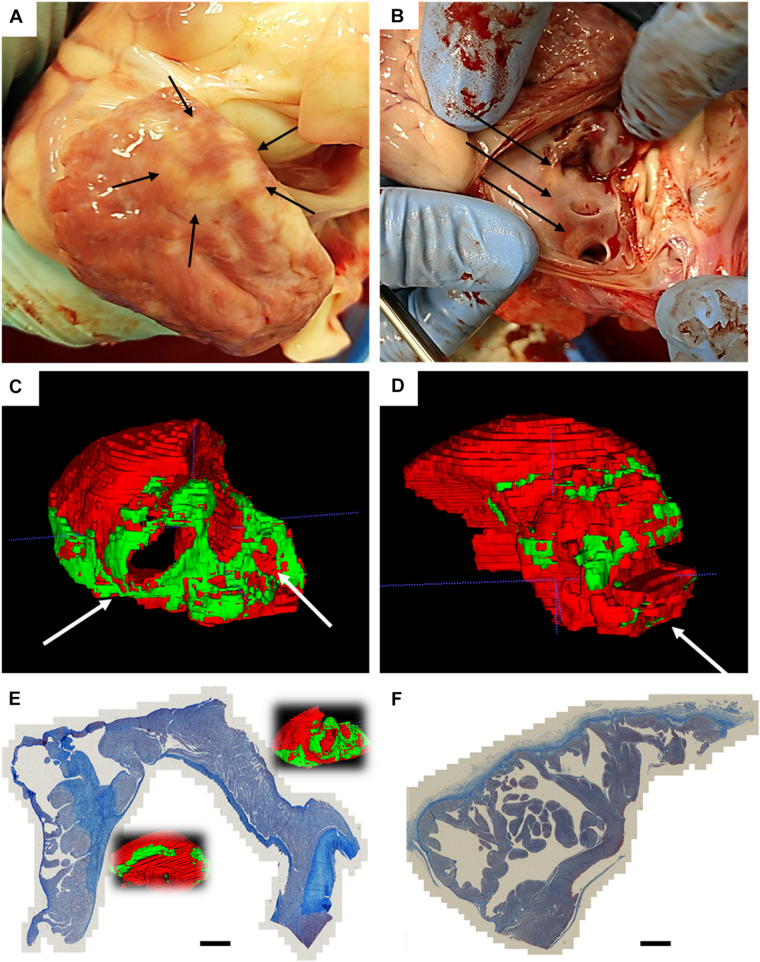
Atrial findings at the end of follow-up. **(A)** Epicardial circular discoloration observed on the left atrium; **(B)** diffuse endocardial discoloration in proximity of the pulmonary veins; **(C)** Posterior view of the 3D rendering of the manual segmentation of normal myocardium (red) and non-myocardial tissue (green), with white arrows pointing at pulmonary vein ostia; **(D)** Posterior view of the 3D rendering of an age-matched non-ablated control, segmented with same criteria; **(E)** trichrome staining of left atrium which underwent AC pulsed electric field ablation, showing circular continuous and transmural lesions; **(F)** trichrome staining of age-matched non-ablated control. Scalebar is 3 mm.

In two animals, we performed a follow-up electroanatomical mapping: we observed, both in the left atrium and ventricle, an increased area of low voltage, consistent with tissue remodeling after myocardial ablation. An example is shown in Supplementary Figures 3A,B. After euthanasia, the chest cavity inspections did not show visible collateral damage on the lungs, esophagus, or pericardium. After excision, the left atrium was closely examined: epicardial discoloration was noticed, in one case in full circular shape (Figure 4A); endocardial discoloration was reported close to the pulmonary vein ostia (Figure 4B).

The *ex vivo* magnetic resonance imaging showed localized tissue discoloration and darkened areas in the expected ablation targets. We scanned and segmented an age-matched untreated left atrium to confirm that atrial changes are not due to the physiological presence of epicardial fat or proximity of the mitral anulus (Supplementary Figures 5A–F). In the ventricular scans we observed two types of tissue changes: septal morphological changes, accompanied by discoloration, and left anterolateral darkening (Supplementary Figures 6A–E). Based on position and appearance, we measured the lesions dimensions and assigned the septal discolorations and darkening to AC-PFA, and the anterior-lateral darkening to RF ablations. The manual atrial segmentation (Supplementary Figures 7A,B and Supplementary Video 2) allowed us to estimate the amount of tissue affected in correspondence with the expected position of the loop catheter. A consistent part of the posterior wall in correspondence of the pulmonary veins presented the mentioned tissue changes, as well as localized areas in the lateral and anterior wall (Figure 4C), not observed in the control atrium (Figure 4D). The volumetric ratio of the non-myocardial tissue was 20.25 ± 4.40% for AC-PFA treated atria (*N* = 5, *n* = 40 slices/heart), versus 9.225 ± 6.175% in the healthy control (calculated across *n* = 40 slices), with *p* = 0.005 with one-sample t-test. After subtraction of the control non-myocardial tissue volume, the percentage of tissue volume ablated was 11.03 ± 4.41% (*N* = 5). Individual comparisons were also significant with respect to the control atrium (Table 3). The atrium-specific volume estimator showed that the ablated volume is 1.412 ± 0.672 times (*N* = 5) the internal volumetric reference. We could not confirm atrial tissue changes transmurality from T1-weighted MRI alone. For anatomical reference, the measured thickness across the atria myocardium is 5.85 ± 2.11 mm, with decreasing thickness on the posterior wall. On the few lesions that were isolated, the width was 22.98 ± 2.49 mm and the depth 5.41 ± 0.52 mm – superior to the numerically predicted effective electric field radius, probably as an effect of multiple pulses.

**TABLE 3:** Volumetric ratio of the non-myocardial tissue as calculated from manual segmentation of the 9.4T MRI atrial scans.

	AC-PFA#1	AC-PFA#2	AC-PFA#3	AC-PFA#4	AC-PFA#5	CTL

	Non-myocardial volume percentage					
**Mean ± SD (%)**	70.34 ± 21.98	78.49 ± 16.41	81.77 ± 12.17	82.71 ± 11.36	82.61 ± 12.40	90.77 ± 6.164
***p*-value vs. CTL**	<0.0001	0.0010	0.0075	0.0100	0.0158	NA

Via trichrome staining we confirmed that the darkened areas of corresponded to post-ablation fibrosis: we observed circular continuous lesions near the pulmonary vein ostia, and transmural lesions from smooth to trabeculated zones, in one heart (Figure 4E); the atrial control presented only connective tissue coloration in correspondence of the endocardial and epicardial lining (Figure 4F).

**FIGURE 5:**
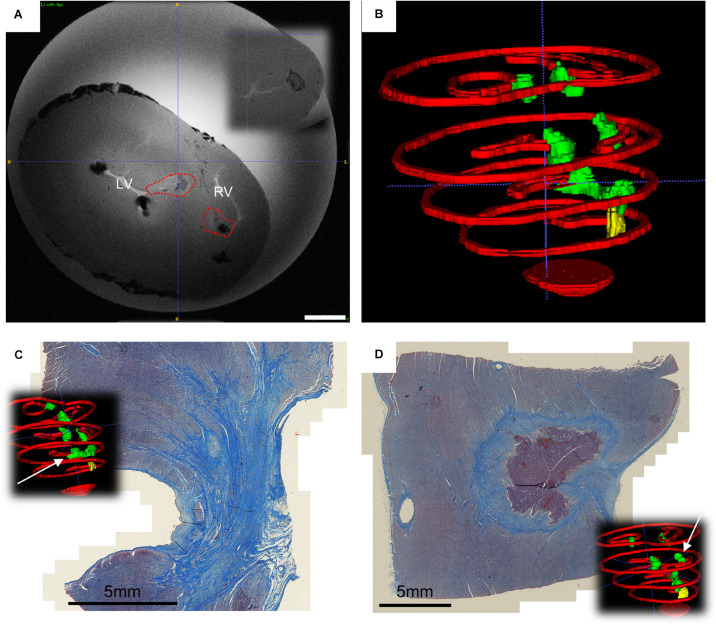
Ventricular findings at the end of follow-up. **(A)** *Ex vivo* T1 axial scans with highlighted changes identified with AC pulsed field ablation lesions (red dotted lines) – in the inset it is visible a control RF ablation lesion. Scale bar is 10 mm; **(B)** Posterior view of the 3D rendering of ventricular myocardial perimeter (red), AC pulsed field ablation lesions (green) and RF ablation lesions (yellow); **(C)** trichrome staining of apical transmural lesions across the interventricular septum; **(D)** trichrome staining of basal isolated AC pulsed electric field ablation lesion, with intact core of cardiomyocytes.

In the ventricles, transmural lesion across the septum could be found at the apical region, whereas isolated opposing lesions were observed as a result of application in more basal locations (Figure 5B and Supplementary Video 3). For reference, the measured septum thickness is 15.21 ± 4.27 mm, increasing toward the base. The lesions linear depth was 5.99 ± 0.75 mm, and the width 6.37 ± 0.62 mm, compatible with the numerically predicted effective radius, despite of misalignment and most probably due to multiple applications.

The trichrome staining confirmed that the observed discolorations were area of undergoing fibrosis, with mixed borders. A remarkable tissue shrinking was observed on transmural lesions (Figure 5C). Interestingly, isolated AC-burst ablation lesions could present a core of intact cardiomyocytes (Figure 5D). The core presents with jagged borders and fibrotic infiltration. No difference could be observed qualitatively between histology of RF lesion and AC-PFA ones (Supplementary Figures 7C,D).

## Discussion

PFA holds the promise to positively disrupt the field of catheter ablation of arrhythmia. This is due to its remarkable differences with respect to RF ablation, specifically: non-thermal nature; tissue specificity; cardiac muscle sensitivity; higher flexibility, speed and versatility. With its benefits, PFA comes with specific safety issues.

In our study, during the AC-PFA applications, we did not estimate a temperature increase able to produce damage. We have validated the non-thermal nature of our AC-burst with electrothermal simulations, finding that temperature increases in tissues are below the thermal damage threshold (Kolandaivelu et al., 2010) and were mitigated in the first tens of milliseconds – 4τ_*tissue*_ = 36.88 ms – after energy delivery. This result is particularly important for atrial applications, were loop catheters with close electrodes imposed electric fields ten times the therapeutic threshold, without the need of irrigation. Accordingly, we did not observe the following issues: temperature increase in the esophagus during atrial applications; charring or clotting on the non-irrigated catheters; lesions on the esophagus during chest inspection. Our findings concur with dedicated animal studies that investigated how PFA does not cause esophageal damage upon direct or endovascularly proximal applications (Neven et al., 2017; Koruth et al., 2020).

Tissue specificity was not an investigative target or our study; nevertheless, we did obtain contained lesions and no observable peripheral damage to the pericardium, esophagus, or lungs. This feature of PFA was extensively investigated in animal models, specifically for coronary vessels (Neven et al., 2014), phrenic nerves (van Driel et al., 2015), and pulmonary veins (van Driel et al., 2014; Witt et al., 2018).

Cardiac muscle sensitivity has been demonstrated in several *in vitro*, *in vivo* (Sugrue et al., 2019), and in pilot clinical studies (Reddy et al., 2018). It represents, with tissue selectivity, the cornerstone of the potential success of PFA in clinical trials. We observed, in atria and ventricles, and efficient ablation by acute changes in electrograms amplitude and shapes, as reported in similar studies (DeSimone et al., 2014; Stewart et al., 2019). Ventricular potential changes in terms of and hyper/depolarization of the baseline were reported in studies characterizing unwanted electroporation during defibrillation (Nikolski and Efimov, 2005). We also report a significant elongation of local electrograms, which has the direct consequence to change the local refractory period of the tissue - although not clearly whether in the absolute or relative refractoriness, nevertheless without elicited atrial or ventricular arrhythmia in presence of amiodarone. After 4 weeks, we observed transmural scars formation both at the atria and the ventricular level. To our best knowledge, we are the first to report the use of purely AC bursts in the left atrium, and particularly in the ventricular endocardium with commercial catheters. A study similar to the one we presented compares the use of symmetrical and asymmetrical – which deeper lesions - biphasic high frequency pulses; several differences exist, among which the use an epicardial unipolar (electrode vs patch) approach (van Es et al., 2019b). We also provide a first use of 9.4T MRI to estimate the tissue changes 4 weeks after non-thermal cardiac ablation. Interestingly, the absence of clear transmural T1 contrast did not implicate the absence of transmural lesions. T1 signal in PFA lesions – which derive from a non-necrotic fibrosis - might be regionally and globally different from RFA ones, and more similar to myocardial signal. Also, due to the spatial gradient of the electric field, the lesion maturation state can differ between endocardial, midmyocardial, and epicardial layers after 4 weeks. Until anatomical contrast-enhanced MRI methods are validated, only histopathology can confirm the extent of chronic lesions after PFA.

The occasional surviving cardiomyocyte core appears preferentially oriented perpendicularly to the cutting plane. It is intriguing to think that the reason of such muscular residual derives from the changing relative angle between the electric field and the cardiac fibers across the septum (Buckberg, 2006). Previous studies demonstrated that in mouse skeletal muscle and ventricular cardiomyocytes the fiber orientation determines different electroporation and irreversible damage levels (Èoroviæ et al., 2010), especially at our employed scale (Semenov et al., 2018). Atrial lesions did not present a different T1 signal or surviving cardiomyocytes in the observed area, but animal studies on intact rabbit hearts hinted that the atria might be more susceptible to electroporation (Fedorov et al., 2008). It is also possible that AC-PFA lesions might take more time to mature, compared to RF ones, due to the non-necrotic scar formation (Reddy et al., 2018).

Flexibility of PFA is demonstrated by the similar efficacy reported with the plethora of pulse features presented in the literature. New pulses or previously unreported applications need to be nevertheless accurately validated, primarily for feasibility and safety, as we presented for symmetrical AC-bursts in this work. Therapies are deliverable from the epicardium or endocardium, in unipolar and bipolar mode. Due to our generator compliance, and in order to prove the efficacy of AC-bursts in a relevant setting, we focused on the endocardial bipolar application. This approach guarantees a tight control on the imposable electric field.

The ablative treatment, with appropriate energy delivery and contact, can be delivered in one shock, but multiple shocks can produce irreversible electroporation effects even at subthreshold levels (Golberg and Rubinsky, 2010; Garcia et al., 2014). Our applications time was below 2 minutes overall and we opted for a multiple AC burst train which proved beneficial in the ventricular settings, were the simulations shows that the area below threshold is expectedly very much dependent on catheter relative placement.

We have presented how PFA methods, and in particular AC-PFA, can readily benefit from the existing catheter technologies: we employed commercially available catheters, in particular non-irrigated diagnostic circular ones, with good efficacy. Other groups, or companies, have presented their own dedicated catheter design (DeSimone et al., 2014; Neven et al., 2014; Reddy et al., 2018; Stewart et al., 2019), which is usually matched with a specific or proprietary PFA generator. The acquired experience within the bioengineering and medical communities led to the overcoming of the initial issues with DC-PFA, such as arching, barotrauma and arrhythmia induction (Guandalini et al., 2019).

Short term safety is related to the effective ECG-gating to synchronize the PFA with the absolute refractory period. Failure to synchronize can potentially trigger lethal (Deodhar et al., 2011). Some PFA generators, like the NanoKnife (Angiodynamics) use an AccuSync R wave trigger (AccuSync MRC) that times the delivery after 50 ms from detection (Bertacchini et al., 2007). Our AC-PFA generator presents a similar lag, with maximum observed delay of 75 ms: this timing is suitable for energy delivery in the safe region of QT intervals ranges (Caluori et al., 2019; Colunga et al., 2019), without myocardial premature excitation. Interestingly, when an occasional synchronization failure occurred, due to ambient electronic noise on ECG leads, the AC-busts delivered on the T-wave did not elicit premature ventricular contractions. This might be due to the high frequency nature of the pulse. Overall, with adequate placement of a return electrode, we did not observe involuntary muscle twitching, but we reported occasional occurrence during single atrial applications.

Long-term safety is an open issue and might be related to electrolytic bubble formation upon DC-PFA applications. This can cause gas microemboli with consequent coronary occlusion, stroke or silent cerebral events. To our best knowledge, there is no systematic characterization of this phenomenon for therapies employing DC-PFA, with a notable exception for LifePak9 DC-pulses which are characterized *in vitro* (van Es et al., 2019a). In our experience, DC pulse trains of microseconds can also produce gas bubble stream (Supplementary Video 4). Therefore, we adopted an AC-burst delivery, which avoids or minimized this issue. Consequently, we did not observe gas bubble stream across the aortic valve, during a trial or ventricular application.

The study presents several limitations: (i) no energy titration was considered, and the generator settings were tuned to the maximum voltage available (ii) the follow-up was limited to 4 weeks and the scarring process (especially in the interventricular septum) might have been interrupted prematurely (iii) the employed circular catheter was selected by diameter for easy maneuverability, and not optimized to obtain an homogeneous electric field – aiming to create a continuous circular scar - along the pulmonary vein ostium (iv) part of the analysis is not blinded for lack of multiple grouping v) only one selected heart was used to fully characterize myocardial changes via both MRI and histology, compelling future works on AC_PFA with systematic histological quantification to test effectiveness and the anatomical relationship between MRI tissue pattern and ablation fibrosis.

## Conclusion

We proved in this chronic animal study that AC-PFA is feasible and safe to induce electrical changes and selectively ablate atrial and ventricular myocardium, with no acute or short-term notable adverse effects. The results support further preclinical investigation of the proposed AC-PFA method with the purpose of atrial and ventricular therapies. The encouraging data on the presence of transmural lesions call for the systematic quantification of lesion formation and features – to estimate effectiveness – and titration of voltage/pulse number combination. For the first time, we proved the feasibility of AC-PFA in ventricular endocardial approaches, although the presence of survived cardiomyocytes constitutes an important warning, needing further characterization and dedicated optimization before therapeutic use.

## Data Availability Statement

The raw data supporting the conclusions of this article will be made available by the authors, without undue reservation.

## Ethics Statement

The animal study was reviewed and approved by University of Veterinary and Pharmaceutical Sciences, Brno.

## Author Contributions

GC designed the study, co-designed the AC pulse generator, supervised the experiments, performed the analysis, interpreted the results, and drafted the manuscript. EO was responsible of the animal protocols, care and samples handling. TJ, MP, and LV assisted in the animal experiments. IP was responsible for the *ex vivo* magnetic resonance imaging. SH designed and performed the numerical simulations. VN and DC co-designed, fabricated and tested the AC pulse generator, and supervised the technical aspect of the animal experiments. VR, AH, and MC secured the use of histological and veterinary facilities. ZS secured funding, approved the animal protocols, the experimental design, and performed the ablation procedures. All authors participated in the manuscript revision.

## Conflict of Interest

SH was employed by BIOTRONIK SE & Co. KG. The remaining authors declare that the research was conducted in the absence of any commercial or financial relationships that could be construed as a potential conflict of interest.

## Abbreviations

AC: alternating current
DC: direct current
PFA: pulsed field ablation
RF: radiofrequency.
