# Supplementary material for: AC Pulsed Field Ablation Is Feasible and Safe in Atrial and Ventricular Settings: A Proof-of-Concept Chronic Animal Study
Source: Front Bioeng Biotechnol. 2020 Dec 3;8:552357. doi: 10.3389/fbioe.2020.552357 (PMC7744788; doi:10.3389/fbioe.2020.552357)
Supplement: Supplementary file 1 [file Table_1.DOCX]

Supplementary Material

# Supplementary methods

*Ex vivo magnetic resonance imaging*

The parameters of the experiment for axial direction images were set to:

- TR = 8562.2 ms,
- TE = 11 ms,
- echo train length (ETL) = 8,
- field of view (FOV) = 110 mm x 110 mm,
- image resolution = 256 x 256,
- 5 averages,
- number of slices = 80,
- slice thickness = 1 mm; no gap between slices.

For sagittal direction scans, parameters of the experiment were set to:

- TR = 10702.8 ms,
- TE = 11 ms,
- ETL = 8,
- FOV = 110 mm x 110 mm,
- image size = 256 x 256,
- 5 averages,
- number of slices = 100,
- slice thickness = 1 mm; no gap between slices.

For coronal direction scans, parameters of the experiment were set to:

- TR = 9632.5 ms,
- TE = 11 ms,
- ETL = 8,
- FOV = 110 mm x 110 mm,
- image size = 256 x 256,
- 5 averages,
- number of slices = 90,
- slice thickness = 1 mm; no gap between slices.

The scans covered the whole volume of the samples. After first scanning of whole hearts, all samples were cut into two parts; the atria part and the ventricle part, which were imaged one more time separately. The procedure of the measurement was repeated as in the first scanning experiment and the same volume coil 1H 198/154 mm was used.

T2-weighted images were obtained from the atrial parts in coronal and axial direction using a TurboRARE sequence with following parameters:

1. coronal scans:
   - TR = 5351.4 ms,
   - TE = 11 ms,
   - ETL = 8,
   - FOV = 90 mm x 90 mm,
   - image size = 256 x 256,
   - 8 averages,
   - number of slices = 50,
   - slice thickness = 1 mm; no gap between slices.
2. axial direction:
   - TR = 8562.2 ms,
   - TE = 11 ms,
   - ETL = 8,
   - FOV = 50 mm x 90 mm,
   - image size = 256 x 256,
   - 8 averages,
   - number of slices = 80,
   - slice thickness = 1 mm; no gap between slices.

T1-weighted images were obtained from the atrial parts in axial direction only using a T1-FLASH sequence with following parameters:

- TR = 1277.7 ms,
- TE = 4.435 ms,
- FOV = 90 mm x 90 mm,
- image size = 512 x 512,
- 4 averages,
- number of slices = 40,
- slice thickness = 1 mm; no gap between slices.

Ventricular sections were scanned afterwards. T2-weighted images were obtained in axial direction only using TurboRARE sequence:

- TR = 9632.5 ms,
- TE = 11 ms,
- ETL = 8,
- FOV = 100 mm x 100 mm,
- image size = 256 x 256,
- 5 averages,
- number of slices = 90,
- slice thickness = 1 mm; no gap between slices.

T1-weighted images were obtained in axial direction using T1-FLASH sequence:

- TR = 1427.5 ms,
- TE = 4.435 ms,
- ETL = 8,
- FOV = 100 mm x 100 mm
- image size = 256 x 256 and 512 x 512,
- 4 averages,
- number of slices = 90,
- slice thickness = 1 mm, no gap between slices.

# Supplementary Tables

**Supplementary Table 1: Material parameters used in FEM simulations**

| **Material** | **Electrical**  **Conductivity**  **f < 1 MHz**  **(S/m)** | **Relative**  **Permittivity**  **@ 1MHz** | **Thermal**  **Conductivity**  **(W/mK)** | **Specific Heat**  **Capacity**  **(J/kgK)** | **Mass Density**  **kg/m^3^** |
| --- | --- | --- | --- | --- | --- |
| Blood | 0.7^1^ | 1000^2^ | 0.54^3^ | 4180^1^ | 1000^1^ |
| Cardiac Tissue | 0.4^1^ | 1000^2^ | 0.7^3^ | 3200^1^ | 1200^1^ |

**Supplementary Table 2: Detailed results of the interventricular septum ablation simulations**

| **Ventricular Model** | **Electric field mean (V/cm)** | **Electric field max LV (V/cm)** | **Electric field max RV (V/cm)** | **Treatment radius (mm)** | **Untreated region length (mm)** |
| --- | --- | --- | --- | --- | --- |
| Perfect alignment | 1945 | 2.36 | 4.74 | 5.1 | 4.3 |
| 10 mm misalignment | 1634 | 2.81 | 4.65 | 4.3 | 8.27 |
| 45° tilting | 1857 | 2.38 | 4.70 | 4.5 | 4.31 |
| Misalignment +tilting | 1668 | 2.32 | 4.55 | 5.0 | 8.35 |

#
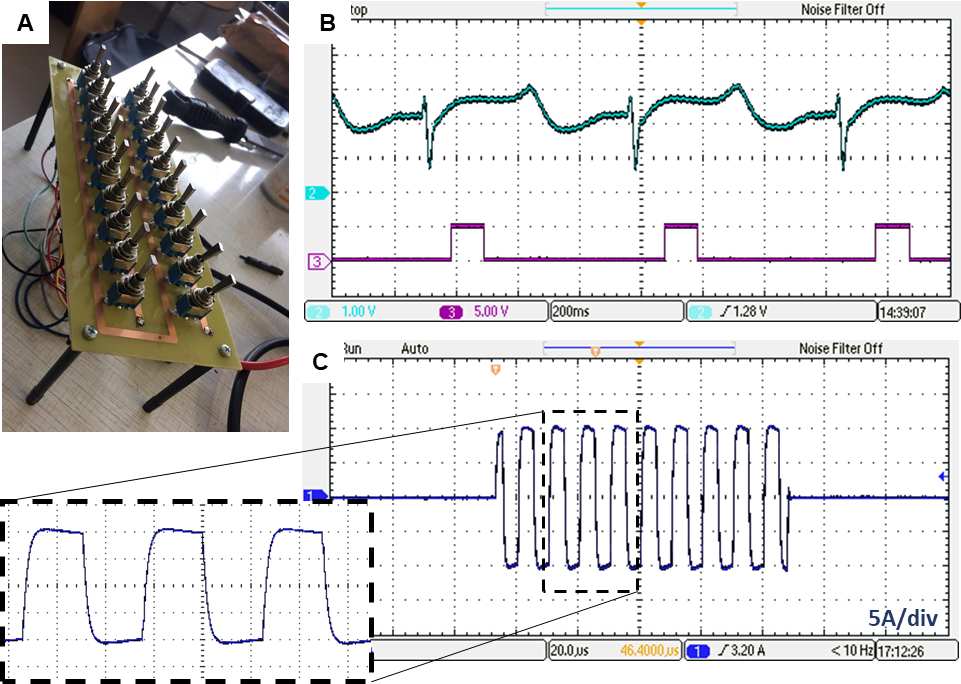
Supplementary Figures

**Supplementary Figure 1: Details of the AC-PFA setup and output.** A) Switchbox used to connect the catheter electrodes to the hot and cold pole of the AC-PFA generator; B) Oscillogram of ECG-gated pulse delivery. The purple signal is a TTL control signal generated by the generator control unit upon burst delivery; C) Example of AC-burst current signal delivered by the generator. The inset presents a zoom of the square wave, showing no upshoot.

**
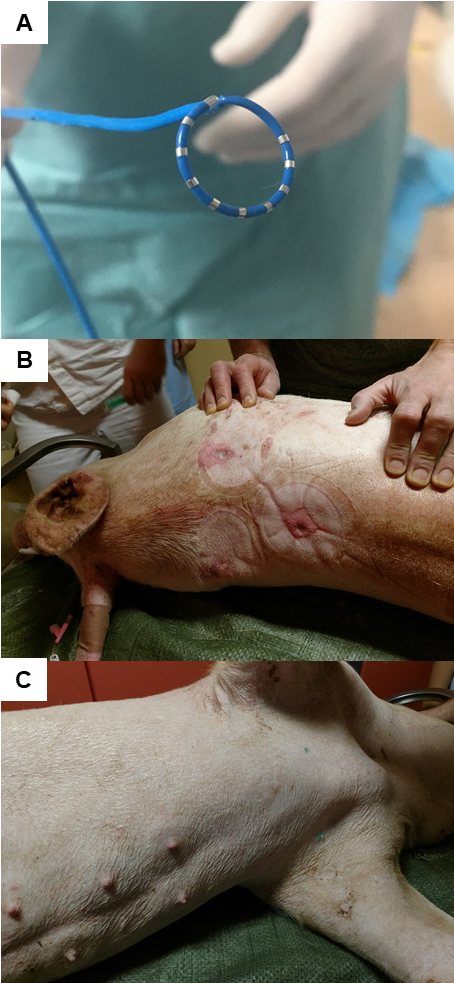
**

**Supplementary Figure 2: Postprocedural findings.** A) Clean circular catheter after extraction; B) back burning localized at the mapping patches site, due to parasitic currents flowing through the operating table. The burns were avoided by adding one more return electrode; C) missing burned area on the chest, confirming a parasitic circuit.


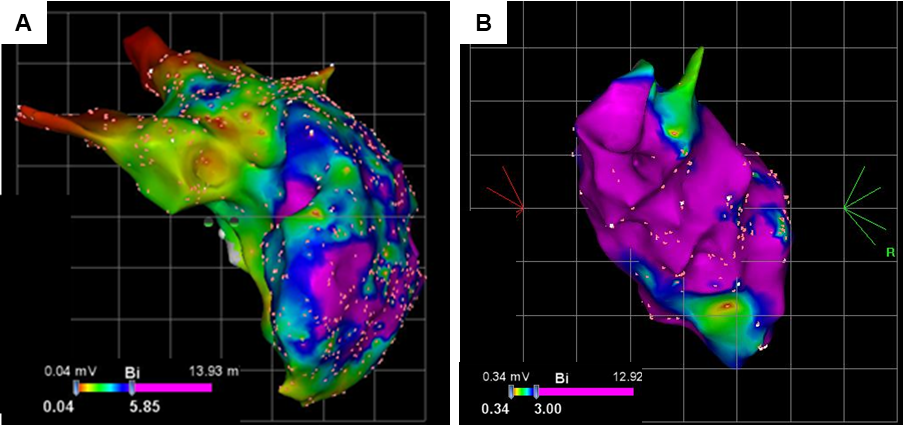


**Supplementary Figure 3: Electroanatomical findings at follow-up.** A) Right-anterior view of the left ventricle of a selected animal, showing increased and diffuse areas of low voltage. B) Right lateral view of the left ventricle of a selected animal, where an apico-septal low voltage area is observed at the previous AC-PFA application area.

**
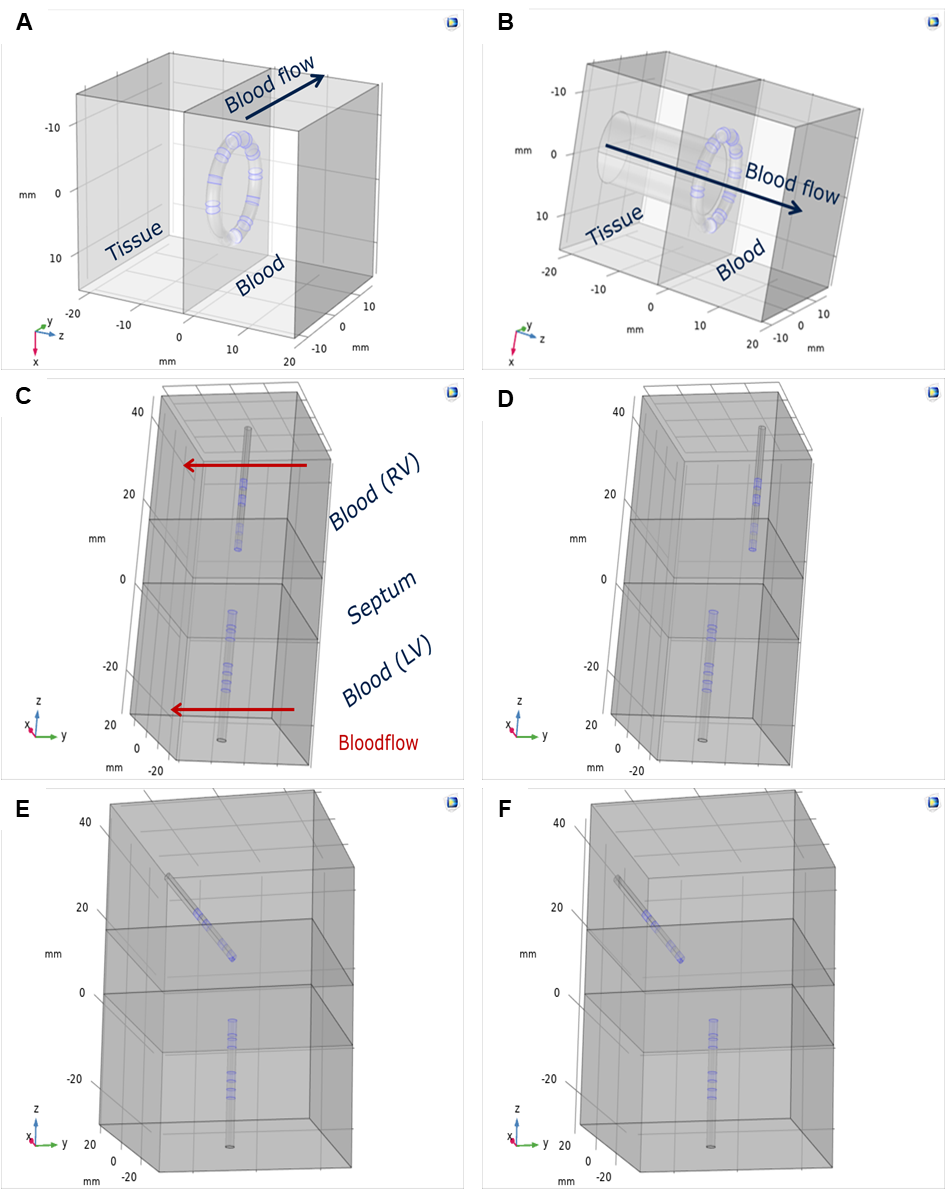
**

**Supplementary Figure 4: Set of simulated geometries.** A) Atrial wall; B) pulmonary vein ostium; C) Interventricular septum with perfect alignment; D) Interventricular septum with 10 mm misalignment; E) Interventricular septum with 45° tilting; F) Interventricular septum with tilting and misalignment.


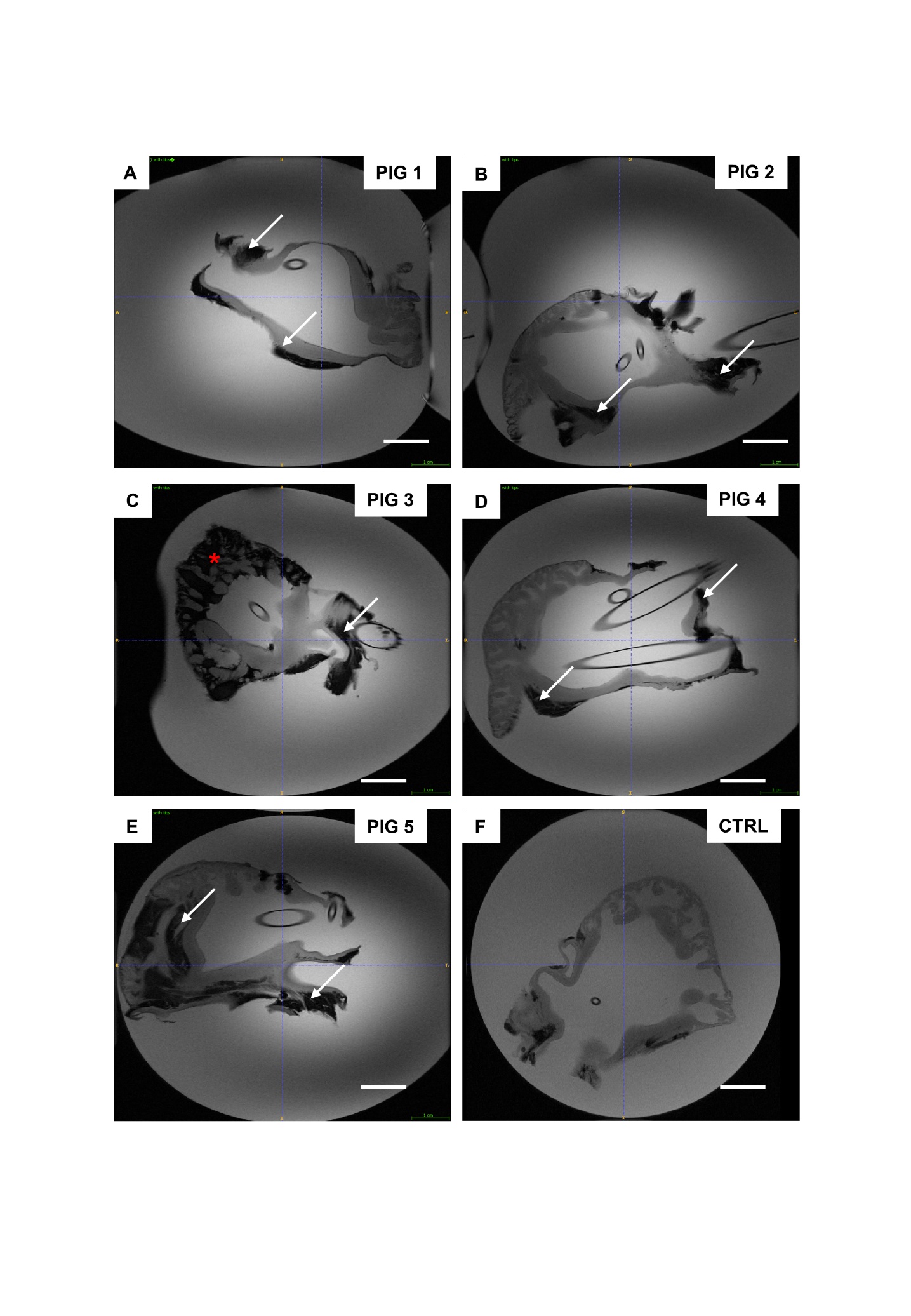
**Supplementary Figure 5: MRI of atrial samples.** A-E) 9.4T atrial scans (T1-weighted) diffuse discoloration and localized darkened areas (white arrows); F) Atrial scan of an age-matched healthy atrium. Scalebar is 10 mm. The red asterisk indicates entrapped blood to avoid confusion with tissue changes.


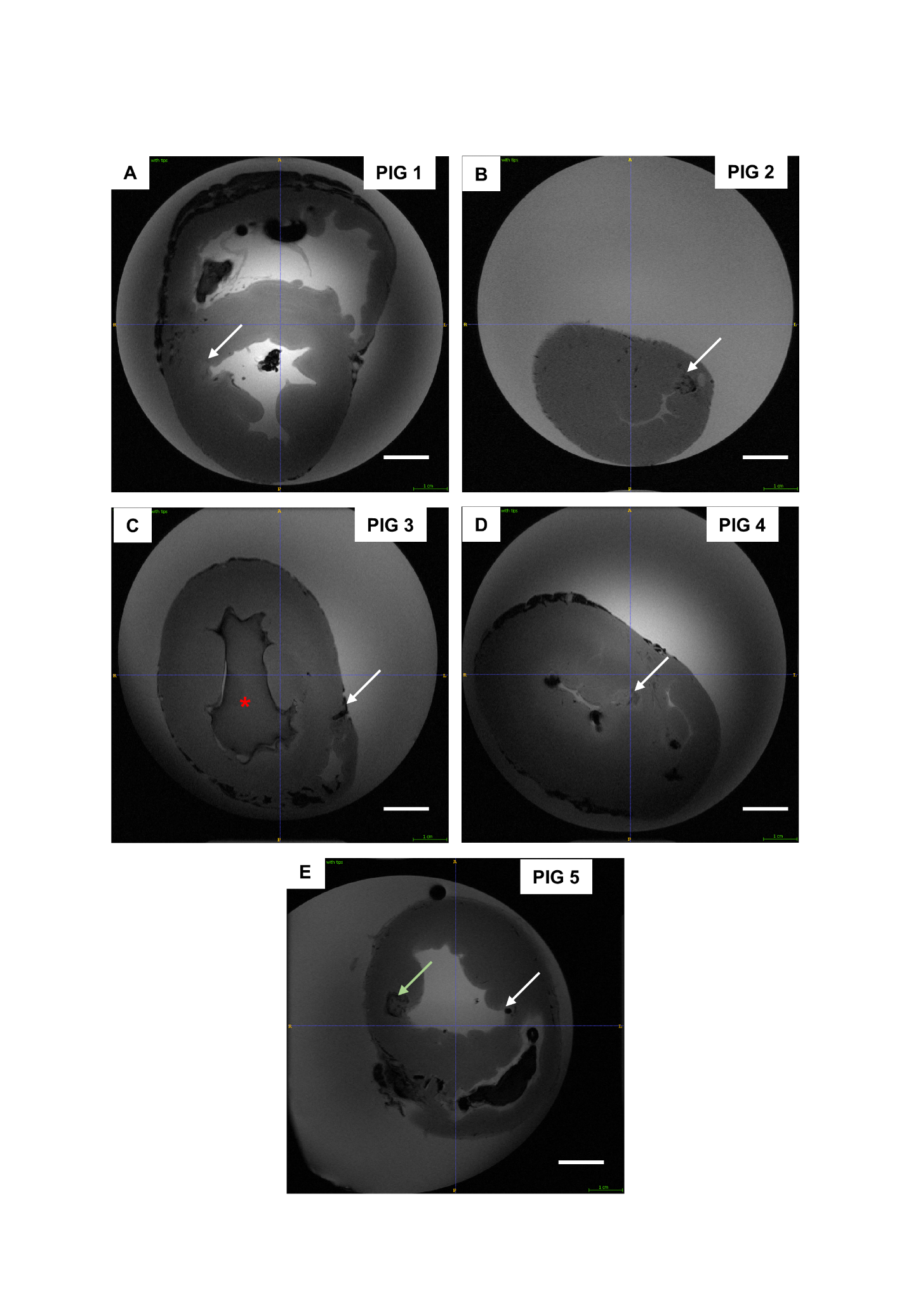
**Supplementary Figure 6: MRI of ventricular samples.** A-E) 9.4T ventricular scans (T1-weighted) showing localized discoloration and darkened areas (white arrow indicates AC-PFA lesions, green arrow radiofrequency lesions)/ Scalebar is 10 mm. The red asterisk indicates entrapped blood.

**
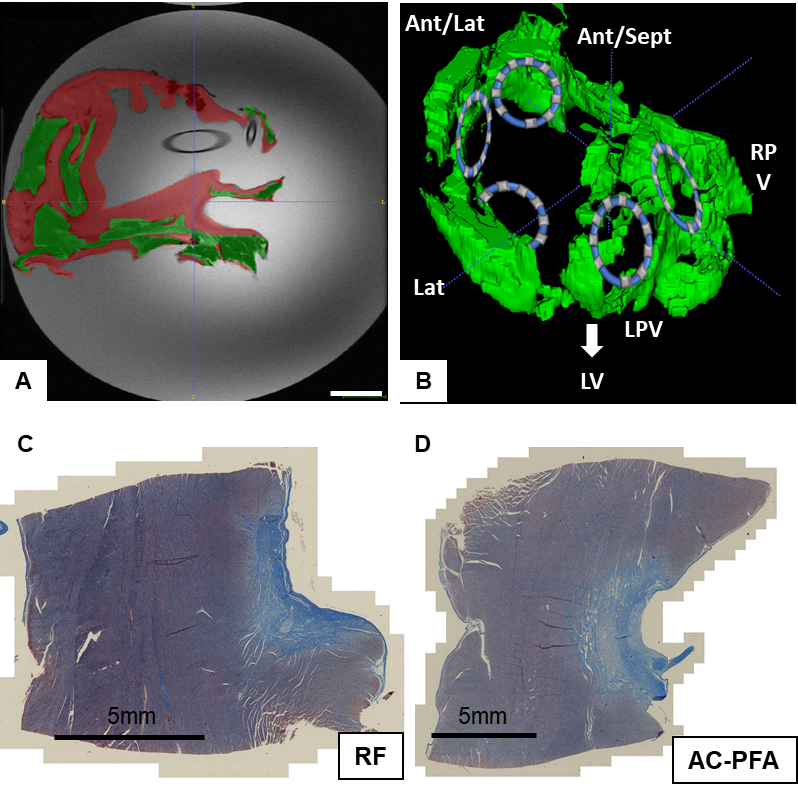
**

**Supplementary Figure 7: Details of tissue analysis.** A) 9.4T atrial scans (T1-weighted) showing masks for segmentation in the atrium (the oval shape is a plastic tip to keep pulmonary veins open). Red=myocardium and green=non-myocardium. Scalebar is 10 mm; B) 3D view of the tissue changes areas that estimates the lesions positions and size on MRI. The view is annotated with the target orientations and the approximate position of the loop catheter at the time of ablation (LPV=left pulmonary vein; RPV=right pulmonary vein; LV=left ventricle). C) Radiofrequency (RF) ablation control lesion on the anterior wall of the left ventricle; D) AC-pulsed field ablation (PFA) isolated superficial lesion in the basal interventricular septum.

# Supplementary References

1. Gabriel, C., Peyman, A. & Grant, E. H. Electrical conductivity of tissue at frequencies below 1 MHz. *Phys Med Biol* **54**, 4863–4878 (2009).

2. Gabriel, C. Compilation of the Dielectric Properties of Body Tissues at RF and Microwave Frequencies. in (1996). doi:10.21236/ada303903.

3. Suárez, A. G., Hornero, F. & Berjano, E. J. Mathematical Modeling of Epicardial RF Ablation of Atrial Tissue with Overlying Epicardial Fat. 9.
